# Mitochondrial Genome Evolution, Genetic Diversity, and Population Structure in British Water Voles (*Arvicola amphibius*)

**DOI:** 10.3390/genes12020138

**Published:** 2021-01-21

**Authors:** Corey Kirkland, Marta Farré

**Affiliations:** School of Biosciences, University of Kent, Canterbury, Kent CT2 7NJ, UK; clk21@kent.ac.uk

**Keywords:** mtDNA, Arvicolinae, water vole, phylogenetics, population genetics

## Abstract

The European water vole (*Arvicola amphibius*) is a rodent within the subfamily Arvicolinae. In Britain, water voles have declined rapidly during the last century, making them a conservation priority. The relationship of *Arvicola* to other genera within Arvicolinae remains debated. Additionally, the impact that captive breeding programs in Britain are having on the genetic diversity of water voles is unknown. We use available mitochondrial genomes to construct the phylogeny of species within Arvicolinae, followed by sequencing the mitochondrial DNA control region of 17 individuals from a captive population of water voles in Britain to assess their genetic diversity and population structure. Our study first provides an updated phylogenetic tree of Arvicolinae using the mitochondrial genome of 31 species. Second, our results show considerable genetic diversity in the captive population of water voles, when compared with natural populations in Britain. We confirm the grouping of British water voles into two clades, with all captive individuals found in the English/Welsh clade. Moreover, captive water voles clustered closely with populations in the South East and East of England. The mitochondrial genome provides a useful marker to study the phylogenetics of this rodent clade and in addition, our study provides support for the breeding program at Wildwood Trust and provides a framework for future conservation genetics studies in this species.

## 1. Introduction

Rodents (order Rodentia) are one of the most speciose orders within the mammalian kingdom, containing around 2552 species and 513 genera [1]. Within Rodentia are the family Cricetidae and the subfamily Arvicolinae (containing voles, lemmings, and muskrats). The subfamily contains 151 extant species and 28 proposed extant genera [2]. However, the exact number of genera is currently debated due to an unresolved phylogeny.

Several studies have aimed to resolve the phylogeny of animals within this clade by using mitochondrial and nuclear markers. Early phylogenetic studies sequenced and analysed the mitochondrial cytochrome *b* (Cyt*b*) gene but found rapid, near-simultaneous radiations when using this marker (reviewed in [3]). This led to the hypothesis that substitution saturation had occurred in this gene in Arvicolinae, reducing its value as a phylogenetic marker. Later studies validated this claim and found that genetic saturation occurred at both transitions and transversions of the Cyt*b* gene in arvicoline species [4].

Subsequent studies used multiple markers to attempt to resolve phylogeny. One study combined mitochondrial Cyt*b* gene, nuclear growth hormone receptor (*GHR*) gene, and morphological characters [3]. Another study sampled 900 Muroidea species, including substantial numbers of arvicolines, using a total of five nuclear markers (*BRCA1*, *GHR*, *Rbp3*, *RAG1*, and *Acp5*) and mitochondrial Cyt*b* [5]. However, all studies were unable to fully resolve the phylogeny of all genera within Arvicolinae due to insufficient taxon sampling and phylogenetic marker selection. Recently, mitochondrial genomes have been sequenced for a small number of Arvicolinae species (e.g., [6,7,8,9,10]. A broader representation of taxa from all genera and an increase in genetic information, either nuclear or mitochondrial genomes, is needed to resolve the phylogeny of this clade. A resolved phylogeny for Arvicolinae is vital for conservation efforts of declining species, in order to define taxonomic units.

One rodent in particular, the European water vole (*Arvicola amphibius*), has seen a rapid decline in the last century in Britain. An increase in predation by the invasive American mink (*Neovision vison*), habitat loss, and pollution of watercourses has had a negative impact on populations across the island. This has led water voles to become a conservation priority in Britain, with several projects seeking to increase numbers and manage suitable habitat. Wildwood Trust (a wildlife park near Canterbury in the South East of England) offers mitigation services, a captive breeding program, and reintroductions of water voles back into the wild. 

Population genetics studies have used the mitochondrial DNA (mtDNA) control region to determine the population structure and genetic diversity of water voles in Britain, with the first study showing that two main haplogroups exist [11]. One haplogroup contains modern English and Welsh populations, whilst the other contains modern Scottish populations, with a minimum of 16 mutational steps between the two.

Analysis of the mtDNA control region of ancient museum specimens, both before the last glacial maximum (the Pleistocene period) and following the Younger Dryas (the Holocene period), found that water voles colonised Britain twice [12]. The first colonisation event occurred before the last glacial maximum (LGM), whilst the second event occurred after. This caused the first colonisers to be displaced north into Scotland, whilst the second colonisers remained in England and Wales, revealing the population structure seen today. Modern populations in both haplogroups were found to have significant genetic structure [11].

In the Southeast and East of England populations of natural water voles showed considerable haplotype diversity and substantial genetic structure between watershed populations, and finer-scale structure between populations within watersheds, when using the mitochondrial DNA control region and microsatellite markers of natural populations [13,14]. However, a detailed study on captive populations of water voles is missing, hindering the outcomes of current conservation efforts.

Here, we used available mitochondrial genomes to further understand the evolution of genera within the rodent subfamily Arvicolinae and the position of genus *Arvicola*, before focusing down on British water voles (*A. amphibius*) using the mtDNA control region. We sequenced 17 captive water voles using a combination of non-invasive and non-destructive genetic sampling methods and then compared genetic diversity and population structure between captive and natural populations in Britain to identify whether genetic diversity was being maintained in captivity and the implications of local reintroductions on natural populations. Our study has applications to both the phylogenetics of Arvicolinae and the conservation of British water voles.

## 2. Materials and Methods

### 2.1. Arvicolinae: Mitochondrial Genomes and Sampled Taxa

All available mitochondrial genomes for Arvicolinae taxa were obtained from databases of the ‘National Centre for Biotechnology Information (NCBI) Nucleotide’ and ‘NCBI BLAST’ (see Supplementary Table S1 for taxa and accession numbers). Outgroup taxa were chosen from the Cricetidae family, the closest family to Arvicolinae [15]. Complete mitochondrial genomes were available from three subfamilies of the Cricetidae family—Cricetinae (*Cricetulus griseus*), Neotiminae (*Peromyscus polionotus*), and Sigmodontinae (*Sigmond hispidus*). One additional outgroup taxon was chosen from the Muroidea family Muridae (*Mus musculus*) and was used to root each of the phylogenetic trees.

### 2.2. Arvicolinae: Phylogenetic Analysis of Mitochondrial Genomes

DNA sequences were aligned using the R package ‘Ape v5.3’ [16] and the ‘clustalomega()’ command, with default parameters. The alignment was trimmed based on gaps on the borders of the alignment. A maximum-likelihood (ML) phylogenetic tree was then constructed using the R packages ‘Phangorn v2.5.5’ [17], Ape v5.3 [16], and ‘ggtree v2.0.2’ [18]. The best nucleotide evolution model was selected using ‘modelTest()’ and ‘bootstrap.pml()’ was used to perform the bootstrap analysis (1000 replicates with optimised topology). PartitionFinder [19,20] was then used to estimate better fitting models for the 13 coding genes in the mtDNA, and for the different codon positions. MEGA X [21] was used to align and concatenate gene sequences, and the previously described R packages and RAxML [22] were used to construct the ML phylogenetic trees. All phylogenetic trees were rooted on the outgroup taxon.

### 2.3. Arvicola amphibius: Sample Collection, DNA Extraction, and Amplification

A total of 20 water voles were sampled at Wildwood Trust (Canterbury, England) on several occasions (Table S2). Four samples were collected from tail tissue of deceased water voles and stored at −20 °C. Six of the samples were hair tufts collected in 2019 and were stored in paper envelopes at room temperature. Another 10 samples were collected in 2019 from faecal pellets found in water bowls within enclosures and were stored at −20 °C. All individuals were randomly chosen, and non-invasive sampling was prioritised. Faecal samples were only collected from enclosures containing single voles or those containing mother and offspring.

DNA was extracted from approximately 1–2 cm of tail tissue using an optimised DNA extraction protocol. Tissue was incubated overnight at 56 °C on a shaking platform in 500 µL of extraction buffer [23] and 20 µL of proteinase K (20 mg/µL). An equal volume of phenol:chloroform:isomyl was added and placed on a rocking platform at room temperature for 30 min. Samples were centrifuged for 5 min at maximum speed and then the upper aqueous phase was transferred to a sterile Eppendorf. DNA was precipitated with an equal volume of isopropanol and centrifuged at 13,000 g for 30 min at 4 °C. Isopropanol was discarded and the DNA pellet washed with 70% ethanol. Pellets were dried and DNA was dissolved in 100–200 µL of ddH_2_O.

For hair samples, a tuft of hair was added to a falcon tube with 680 µL of extraction buffer [24] and 80 µL of proteinase K (20 mg/µL) and then incubated overnight at 56 °C with agitation. An equal volume of buffer was then aliquoted into two Eppendorf’s before following the previously described optimized protocol above. DNA was dissolved in 50 µL of ddH_2_O.

DNA was extracted from faecal samples using ‘Qiagen QIAamp DNA Stool Mini Kit’ or ‘Qiagen QIAamp PowerFecal DNA Kit (Hilden, Germany)’ following the manufacture’s protocol. The former method eluted 100 µL of Qiagen buffer ATE, whilst the latter eluted 75 µL of ATE. DNA was precipitated using 3M Na-acetate (pH 5.2) and 100% ethanol, with an overnight incubation at −20 °C, followed by two washes with 70% ethanol. DNA was resuspended in 50 µL of ddH_2_O.

Forward and reverse primers for the mitochondrial DNA control region were selected from a previous publication and were F 5′-TTAATCTACCATCCTCCGTGAAACC-3′ and R 5′-TKGACACTGGTCTAGGGATATTTGC-3′ [11]. All 20 samples were amplified using a PCR reaction mix containing 1× PCR buffer, 200 µm of each dNTP, 0.5 µm forward primer, 0.5 µm reverse primer, and 2.5 units/reaction ‘Qiagen HotStarTaq DNA Polymerase’. Template DNA was then added (9–47 ng/µL). A 15-min denaturation stage was required at 95 °C, followed by 35 cycles (94 °C for 1 min, 50 °C for 1 min, and 72 °C for 1 min), ending with 10 min at 72 °C. A negative control and separate PCR workstation were used for the preparation of the PCR reaction mix to prevent contamination. Amplification was assessed by gel electrophoresis (1% agarose gel, 90 V, ~60 min, and viewed with Syngene Gel Doc). Samples were purified using ‘Qiagen QIAquick PCR Purification Kit’(Hilden, Germany) following the manufacture’s protocol. DNA concentrations and purity were measured following PCR clean-up using NanoDrop (Wilmington, DE, USA).

The amplified DNA was sent for sequencing at DBS Genomics, Durham, UK. Both the forward and reverse strands for each individual water vole were sequenced. A total of 17 out of 20 sequences were successfully amplified. The package ‘Geneious Prime 2020.1 (https://www.geneious.com)’ was used to create a consensus sequence from both strands of DNA. This included reverse complementing the reverse strand and subsequently aligning both strands using the global alignment tool, with free gaps and 93% similarity. Consensus sequences were exported as FASTA files.

### 2.4. Arvicola amphibius: Haplotype Networks and Phylogenetic Analysis

The 17 mitochondrial DNA sequences from Wildwood Trust were aligned, along with sequences from selected papers [11,12,14] following the methods described in Section 2.2 (Table S3). Haplotypes were computed from the multiple sequence alignment results using the R package ‘Pegas v0.13’ [25] with the ‘haplotype()’ command and haplotype networks were computed using the ‘haploNet()’ command, both with default parameters. A MLphylogenetic tree was constructed using all mitochondrial DNA control region sequences following the methods described previously.

### 2.5. Arvicola amphibius: Population Genetics Statistics

Nucleotide diversity, haplotype diversity, and Tajima’s D were calculated in the R package ‘Pegas v0.13’ [25] using multiple sequence alignment results containing different individuals based on location and age. MtDNA control region sequences of *Myodes glareolus* were used as a comparison to *A. amphibius*. All sequences were obtained from [26,27] and multiple alignments were computed based on location, before calculating population genetics statistics for each.

## 3. Results

### 3.1. Phylogenetics of Arvicolinae

All available mitochondrial genomes from NCBI were firstly aligned and trimmed producing a 16,557 bp alignment with 7851 sites with at least one substitution and 35 taxa (31 Arvicolinae taxa and four outgroup taxa). A ML phylogenetic tree was constructed using the generalised time reversible (GTR) + G + I substitution model, based on the lowest Akaike information criterion (AIC) value (Figure S1).

**Figure 1:**
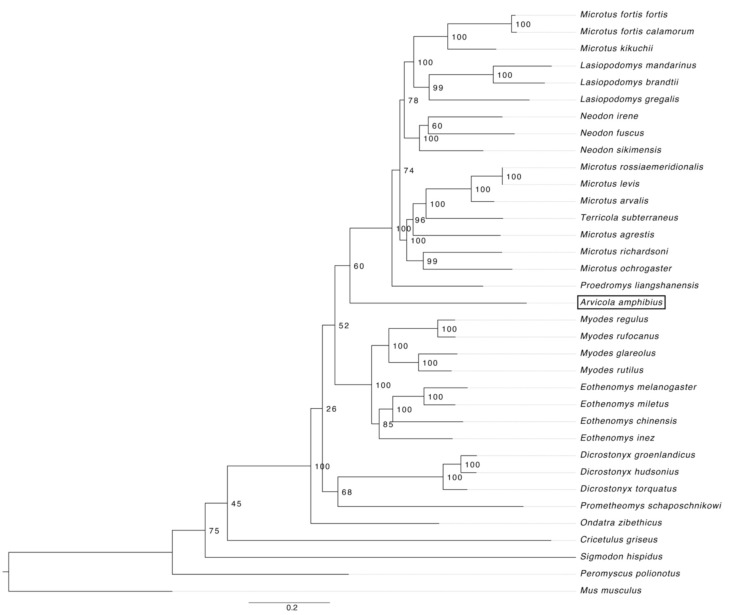
The maximum-likelihood (ML) phylogenetic tree of all available Arvicolinae species based on 13 protein-coding mitochondrial genes, rooted on the outgroup taxon *Mus musculus*.

To improve support, all 13 mtDNA protein-coding gene alignments were concatenated and PartitionFinder was used with two partitions grouping first and second codon position independently to third codon position, with GTR + G + I chosen as the best substitution model for both partitions. The output was then used to construct a ML phylogenetic tree using RA×ML (Figure 1). 

The ML phylogenetic tree for Arvicolinae had high bootstrap scores for nodes within genera, but lower bootstrap scores (below 50%) for nodes between genera (Figure 1). The four outgroup species all diverged first before Arvicolinae taxa, with the tree rooted on *M. musculus*. Genera *Ondatra* diverged first within Arvicolinae, followed by genera *Dicrostonyx* and *Prometheomys* forming a monophyletic clade. *Myodes* and *Eothenomys* species diverged next (clade ‘Clethrionomyini’) and formed a monophyletic group. The remaining genera formed the clade ‘Arvicolini’. Genus *Arvicola*, containing *A. amphibius*, diverged first, followed by genus *Proedromys*. The remaining taxa formed two clades, one containing two *Microtus* species (*M. fortis* and *M. kikuchii*)*, Lasiopodomys*, and *Neodon*, whilst the other contained the remaining *Microtus* spp. and *Terricola subterraneous*.

An additional ML tree of all 13 protein-coding genes in the mtDNA was produced using PartitionFinder to partition by gene and GTR + G + I was identified as the best substitution model (Figure S2). Tree topology was consistent in all three ML trees, except for the position of *Ondatra.*

### 3.2. Population Genetics of Arvicola amphibius

**Figure 2:**
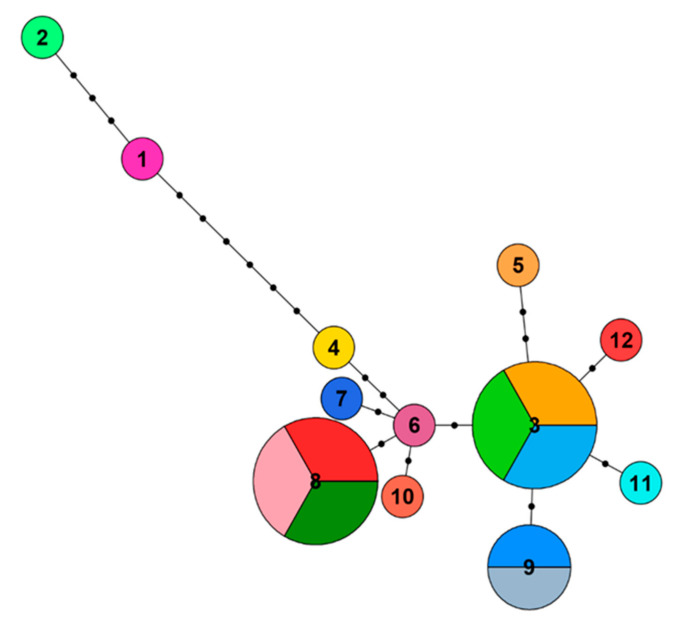
Haplotype network of the mitochondrial DNA control region for 17 captive water voles at Wildwood Trust. Each pie chart represents a unique haplotype and each colour represents a different individual. The dotted lines represent the number of mutational steps between haplotype sequences.

A total of 17 mitochondrial DNA control region sequences were sampled from 17 individual captive water voles at Wildwood Trust and a multiple sequence alignment was computed. The alignment was 706 bp in length and contained 14 sites with a least one substitution. A haplotype network was then constructed using the multiple sequence alignment result (Figure 2). Out of 17 individuals, there were 12 haplotypes and two main haplogroups, with six mutational steps between haplogroups (i.e., between haplotypes 1 and 4). One haplogroup contained haplotypes 1 and 2, and the other contained the 10 remaining haplotypes. Three haplotypes (3, 8, and 9) were shared by more than one individual. Within the largest haplogroup, there was a maximum of two mutational steps between each haplotype.

**Figure 3:**
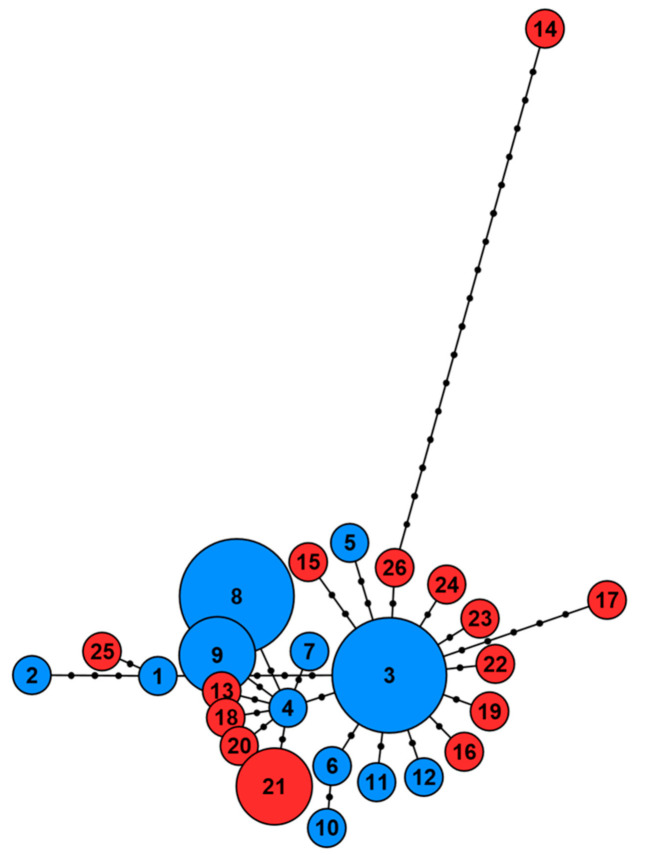
Haplotype network of the mitochondrial DNA control region for 17 captive water voles at Wildwood Trust (blue) and 15 haplotype sequences from natural water vole populations in the South East and East of England (red).

The 17 mitochondrial DNA sequences of captive water voles were aligned with 15 haplotype sequences previously published [14], which were from natural water vole populations in the South East and East of England (Figure 3). The alignment contained 707 sites, with 35 sites with at least one substitution, and resulted in 26 haplotypes (12 captive and 14 natural). The haplotype network formed two haplogroups. One contained only the South East of England haplotype 14 and was 17 mutational steps from the second haplogroup. The second haplogroup contained the remaining captive and natural water vole haplotypes. All captive individuals were found in separate haplotypes to natural water voles. Captive haplotypes 1 and 2 were closely clustered with the East of England haplotype 25, with a minimum of five mutational steps from other haplotypes in the haplogroup (i.e., between haplotypes 1 and 3). Moreover, 14 haplotypes were clustered around the captive haplotype 3, with between one and five mutational step difference, and seven haplotypes were clustered around captive haplotype 4 by one mutational step.

Captive water vole sequences were also aligned to additional mitochondrial DNA control region sequences from natural populations. The samples obtained from these publications ranged in age from recent wild populations to museum specimens dating back to the Pleistocene period. The multiple sequence alignment contained a total of 144 *Arvicola* taxa (*A. amphibius*, *Arvicola scherman*, and *Arvicola sapidus*) and was 642 bp in length, with 117 sites with at least one substitution. A ML phylogenetic tree was then constructed using the generalised time reversible (GTR) + G + I substitution model, based on the lowest AIC value.

**Figure 4:**
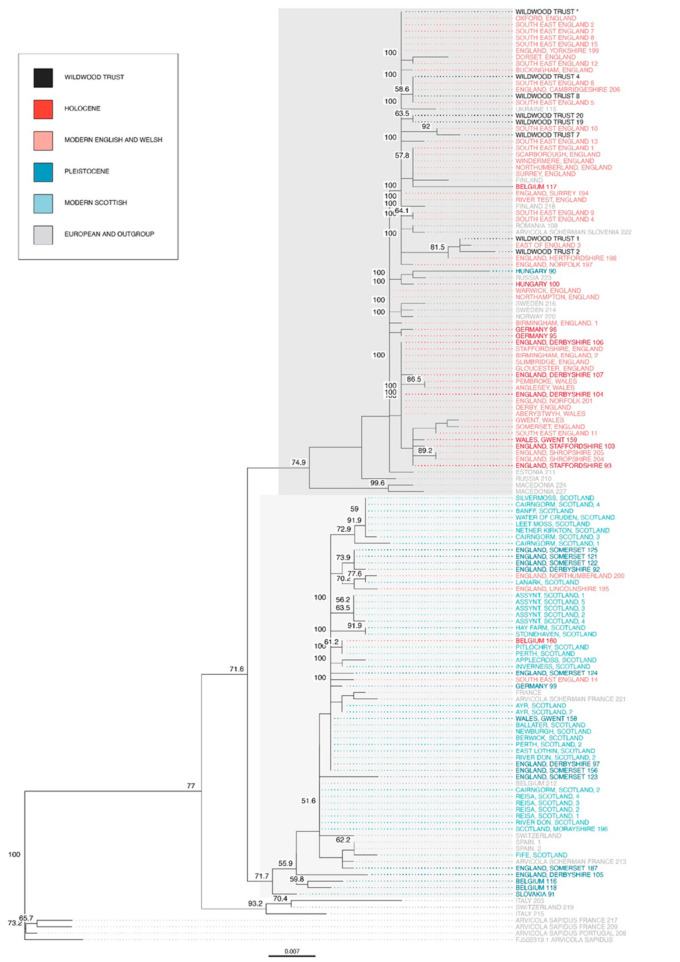
ML phylogenetic tree of sampled *Arvicola* individuals rooted on *Arvicola sapidus*. Taxon colour represents the age or location of the sample. The taxon ‘Wildwood Trust*’ contains multiple Wildwood Trust samples that formed a monophyletic group. Only bootstrap scores greater than 50% are shown.

All Holocene, most modern English and Welsh, all South East and East of England, and all captive Wildwood Trust (England) water voles were grouped into one clade (Figure 4, highlighted in dark grey). The sister clade contained all Pleistocene, all modern Scottish, and three modern English water voles (highlighted in light grey). Samples from mainland Europe were found in both clades. Water voles from Italy and Switzerland were grouped into a separate, early diverging clade after the outgroup taxa (*A. sapidus*). *A. scherman* samples were found in both the English and Welsh clade and the Scottish clade. The phylogenetic tree had relatively high bootstrap scores at nodes separating the major three clades. Polytomies were seen at nodes within the major clades, therefore relationships between individuals were less clear.

### 3.3. Comparison of Genetic Diversity

Population genetics statistics were calculated for each alignment containing mtDNA control region sequences from modern captive water voles, modern natural South East and East of England haplotypes, modern natural English and Welsh water voles, modern natural Scottish water voles, modern natural British water voles, modern natural mainland European water voles, and ancient museum specimens of British water voles (Table 1). 

Wildwood Trust water voles had a lower haplotype and nucleotide diversity (0.949 and 0.004, respectively) compared with natural British water voles (0.971 and 0.016, respectively). Tajima’s D was lower in Wildwood Trust water voles (−2.186) compared with natural British populations (−0.113). Natural English and Welsh water voles had a higher haplotype and nucleotide diversity (0.982 and 0.008, respectively), and a lower Tajima’s D value (−2.164) than natural Scottish water voles which had a haplotype diversity of 0.945, a nucleotide diversity of 0.009, and Tajima’s D of −1.857. Ancient British water voles had a higher haplotype diversity and nucleotide diversity compared with natural British populations (0.993 and 0.017, respectively). Tajima’s D was comparable to natural modern British samples at −0.176.

To put British water vole genetic diversity into perspective, we compared population genetics statistics with another Arvicolinae species, the bank vole (*M. glareolus*), which currently has stable numbers in Britain. Available mitochondrial DNA control region sequences for the bank vole were aligned, and population genetics statistics were computed (Table 1). Focusing on British populations, bank voles had lower haplotype and nucleotide diversity (0.967 and 0.006, respectively) than natural British water voles and a higher Tajima’s D value (−1.775). 

## 4. Discussion

The mitochondrial genome provided a phylogeny with relatively high bootstrap scores at several nodes and no polytomies in the ML phylogenetic tree (Figure 1). Our results support the clade ‘Cletherionomyini’, containing genera *Eothenomys*, *Myodes*, and *Alticola* (not sampled), as well as support for the clade ‘Arvicolini’ containing sampled genera *Arvicola*, *Proedromys*, *Microtus*, *Terricola*, *Lasiopodomys*, and *Neodon*. Moreover, our results confirm the need to reclassify several genera within Arvicolini due to the paraphyletic nature of *Microtus*. The genus *Arvicola*, containing the European water vole (*A. amphibius*), was found to diverge first within the sampled Arvicolini species, followed by genus *Proedromys*. The position of *Ondatra* as the most basal arvicoline differs between the three ML trees described, with low bootstrap scores in two of them. 

Previous publications have found polytomies at major nodes when using individual mitochondrial and nuclear markers to assess the phylogeny of Arvicolinae (e.g., [28]). Studies using multiple mitochondrial or nuclear markers have found more resolution (e.g., [3,5]), whereas more recently available mitochondrial genomes have been used, resulting in more resolved phylogenetic trees, with well-supported nodes (e.g., [6,7,8,9,10]). Our tree topology broadly agrees with a recent study using a 31-nuclear gene supermatrix [15], where Clethrionomyini, containing *Myodes* and *Eothenomys,* form a monophyletic clade, while *A. amphibius* diverged first within Arvicolini species and *Microtus* spp. form a paraphyletic clade. A study constructing the phylogeny of *Microtus* species using the mitochondrial genome and nuclear genotyping-by-sequencing found similar tree topologies in both approaches [29]. Our study further supports the use of mitochondrial genomes as a phylogenetic marker to resolve the phylogeny of arvicoline species. We provide the most recent phylogeny of Arvicolinae using the mitochondrial genome, with the greatest number of sampled species and genera to date. However, to resolve the evolutionary relationships between species in this subfamily fully, new nuclear markers or full genome sequences for as many genera as possible will also be needed. Still, sequencing the mitochondrial genome of an animal remains far cheaper than sequencing nuclear genomes, and by doing so could provide a more resolved phylogeny for this group in the near future.

The highly variable mtDNA control region provides a useful tool to assess the genetic diversity and population structure of water vole (*A. amphibius*) populations. Our study finds considerable genetic diversity in captive water voles at Wildwood Trust. This was based on the haplotype diversity of 17 individuals (Table 1). This population also had a considerable population structure in the constructed haplotype network (Figure 2). Compared with natural populations in Britain, the captive population had maintained haplotype diversity, with a small difference of 0.022 (Table 1). Nucleotide diversity in the captive population was 0.004, in natural English and Welsh water voles was 0.008, and in natural British populations was 0.016, showing a small decrease in nucleotide diversity in the captive population. 

When comparing a declining species in Britain (*A. amphibius*) with a species with stable numbers in Britain (*M. glareolus*), we found a similarity in haplotype diversity, with a difference of 0.004 in the mtDNA control region, whilst nucleotide diversity was lower in bank voles by 0.01. Moreover, it has been found that there is the same ‘Celtic fringe’ genetic pattern in British bank voles as British water voles [26]. Our analysis suggests that although numbers are declining, genetic diversity in British water voles remains high.

In the Southeast and East of England, most captive and natural haplotypes clustered into one haplogroup (Figure 3). Only one natural water vole haplotype, southeast England 14 (Dartford, Kent), diverged considerably from all other haplotypes by a minimum of 17 mutational steps and formed the second haplogroup. This haplotype was shown to cluster with Scottish haplotypes (Figure 4) and may have resulted from reintroductions in the area [14]. Captive haplotypes 1 and 2 diverged with natural haplotypes 25 from the East of England by at least five mutational steps from the remaining haplogroup. This may suggest that these captive individuals were obtained or bred with water voles from the East of England, rather than more locally. Removing these haplotypes from the alignment decreased the haplotype diversity of the captive population to 0.933. But this is still considerably high for a declining population in captivity.

Constructing a phylogenetic tree using all available water vole sequences, including the captive population, natural modern populations, ancient specimens, and sequences from *A. sapidus* and *A. scherman* resulted in three major clades (Figure 4). One clade diverged earlier containing modern Scottish and Pleistocene samples, whilst the other clade diverged later and contained modern English and Welsh, and Holocene samples. This supports previous studies which proposed that modern English and Welsh and modern Scottish water voles had diverged considerably into two distinct haplogroups, as well as supporting the hypothesis that there were two colonisation events into Britain which had shaped the current phylogeographic structure [10,11]. Our study shows that all captive water voles from Wildwood Trust were found in the English and Welsh clade, ensuring any local reintroductions do not cause admixture.

The maintenance of these two British lineages is necessary due to their considerable divergence and their distinct evolutionary history. In the Southeast of England, water vole mtDNA was found to be structured by watershed and it was suggested that conservation management should take place at a local level to maintain local genetic heritage [13]. Ex-situ conservation can be a useful tool to halt the decline of species, especially when genetics and pedigrees are considered. This can avoid inbreeding captive species and therefore limit inbreeding depression. Reintroductions should consider the genetic effects of reintroducing species back into natural populations to ensure distinct genetic features and genetic variability are maintained. These can be lost in reintroductions due to the integration of alien gene pools into natural populations, leading to outbreeding depression and genetic swamping, and a decrease in evolutionary potential [30,31]. This shows the importance of considering genetics in conservation management to maintain genetic diversity and population structure.

## 5. Conclusions

To conclude, our study supports the use of mitochondrial genomes in helping to resolve the phylogeny of Arvicolinae and provides further support for several clades, including the paraphyletic nature of *Microtus* and the position of *Arvicola* as a basal ‘Arvicolini’ genera. Further sequencing of mitochondrial genomes for all genera followed by all species is needed to resolve the subfamilies phylogeny. Our study also shows that the mtDNA control region can be useful in further understanding the genetic diversity and population structure of British water voles. We provide a first insight into the genetic diversity and population structure of captive water voles in Britain and a framework for further study, with a focus on non-invasive and non-destructive genetic sampling. Our results show that the captive population has considerable genetic diversity when compared with natural populations in Britain and are closely related to populations in the Southeast and East of England. In the future, sequencing the whole mitochondrial genome for additional captive and natural populations will provide a more thorough investigation, as will sequencing nuclear genomes to allow for comparisons of heterozygosity and the calculation of fixation indices.

## Figures and Tables

**Table 1:** Population genetics statistics of the mitochondrial DNA control region of European water voles (*Arvicola amphibius*) and bank voles (*Myodes glareolus*). Water vole statistics were calculated based on multiple sequence alignments containing sequences of modern captive water voles, modern natural water voles, and ancient museum specimens of British water voles from the Pleistocene and Holocene periods. Bank vole statistics were calculated using multiple sequence alignments of modern natural bank voles.

*Arvicola amphibius*	n	bp	Hap. No.	Hap. Div.	π	D	P Norm.	P Beta
Captive (Wildwood Trust)	17	706	12	0.949	0.004	−2.186	0.029	0.007
Natural South East and East of England	15	731	14	N/A	0.007	−2.378	0.017	0.000
Natural English and Welsh	32	644	32	0.982	0.008	−2.164	0.030	0.011
Natural Scottish	25	644	25	0.945	0.009	−1.857	0.063	0.041
Natural British	67	639	39	0.971	0.016	−0.113	0.910	0.950
Natural Mainland European	20	639	19	0.995	0.024	−0.753	0.452	0.491
Ancient British	17	634	16	0.993	0.017	−0.176	0.861	0.898
***Myodes glareolus***								
British	24	940	17	0.967	0.006	−1.115	0.265	0.281
All	118	940	97	0.996	0.009	−1.775	0.076	0.050

Abbreviations: Number of sequences (n), trimmed alignment length in base pairs (bp), number of haplotypes (Hap. No.), haplotype diversity (Hap. Div.), nucleotide diversity (π), Tajima’s D (D), and *p*-value for a normal distribution (P Norm.) and beta distribution (P Beta) for Tajima’s D.

## Data Availability

Not applicable.

## Supplementary Materials

The following are available online at https://www.mdpi.com/2073-4425/12/2/138/s1, Table S1: Arvicolinae and outgroup mitochondrial genome accession numbers, Table S2: Sequenced Wildwood Trust water vole samples, Table S3: Additional water vole sequences from previous publications, Figure S1: ML phylogenetic tree of all available mtDNA genomes for Arvicolinae, Figure S2: ML phylogenetic tree of the 13 coding genes in the mitochondrial genome for Arvicolinae.
