# Supplementary material for: Mitochondrial Genome Evolution, Genetic Diversity, and Population Structure in British Water Voles (Arvicola amphibius)
Source: Genes (Basel). 2021 Jan 21;12(2):138. doi: 10.3390/genes12020138 (PMC7910943; doi:10.3390/genes12020138)
Supplement: Supplementary file 1 [file genes-12-00138-s001.pdf]

Supplementary Materials:

Table S1: Arvicolinae and outgroup mitochondrial genome accession numbers.

**Species Name**	**Accession Number**
Lasiopodomys brandtii	MN614478.1
Lasiopodomys mandarinus	JX014233.1
Lasiopodomys gregalis	MN199169.1
Microtus fortis fortis	JF261174.1
Microtus fortis calamorum	JF261175.1
Microtus kikuchii	AF348082.1
Neodon irene	NC016055.1
Neodon fuscus	MG833880.1
Neodon sikimensis	KU891252.1
Microtus rossiaemeridionalis	DQ015676.1
Microtus levis	NC008064.1
Microtus arvalis	MG948434.1
Terricola subterraneus	MN326850.1
Microtus agrestis	MH152570.1
Microtus richardsoni	MT225016.1
Microtus ochrogaster	KT166982.1
Proedromys liangshanensis	FJ463038.1
Arvicola amphibius	MN122828.1
Myodes regulus	NC016427.1
Myodes rufocanus	KT725595.1
Myodes rutilus	MK482363.1
Myodes glareolus	KF918859.1
Eothenomys melanogaster	KP997311.1
Eothenomys miletus	KX014874.1
Eothenomys chinensis	FJ483847.1
Eothenomys Inez	KU200225.1
Ondatra zibethicus	KU177045.1
Dicrostonyx hudsonius	KX683880.1
Dicrostonyx groenlandicus	KX712239.1
Dicrostonyx torquatus	MN792940.1
Prometheomys schaposchnikowi	NC049036.1
Cricetulus griseus	DQ390542.2
Peromyscus polionotus	KY707301.1
Sigmodon hispidus	KY707311.1
Mus musculus	V00711.1

Table S2: Sequenced Wildwood Trust water vole samples.

Sample No.	Sample Type	Enclosure No.	Local ID	Sex
1	Tissue	TB31	-	-
2	Tissue	WW46	-	-
3	Tissue	WW0304/34	-	Male
4	Tissue	WW34/39	-	-
5	Hair	Q88	-	Male
6	Hair	Q100	-	Male
7	Hair	R95	-	Male
8	Hair	R12	-	Male
9	Hair	R28	-	Male
10	Hair	Q100	-	Male
11	Faecal	R2	2228	Male
12	Faecal	Q52	2245	Female
13	Faecal	Q42	2218	Female
14	Faecal	Q7	2264	Female
15	Faecal	Q75a	2326	Female
16	Faecal	R50	2232	Male
17	Faecal	R51	2225	Male
18	Faecal	Q58	2314	Male
19	Faecal	Q100	2185	Female
20	Faecal	R27	2445	Female

Table S3: Additional water vole sequences from previous publications.

Sequence Description	Publication
Modern South East and East of England	Baker 2015
Modern British and European	Piertney *et al.* 2005
Modern and Ancient	Brace *et al.* 2016

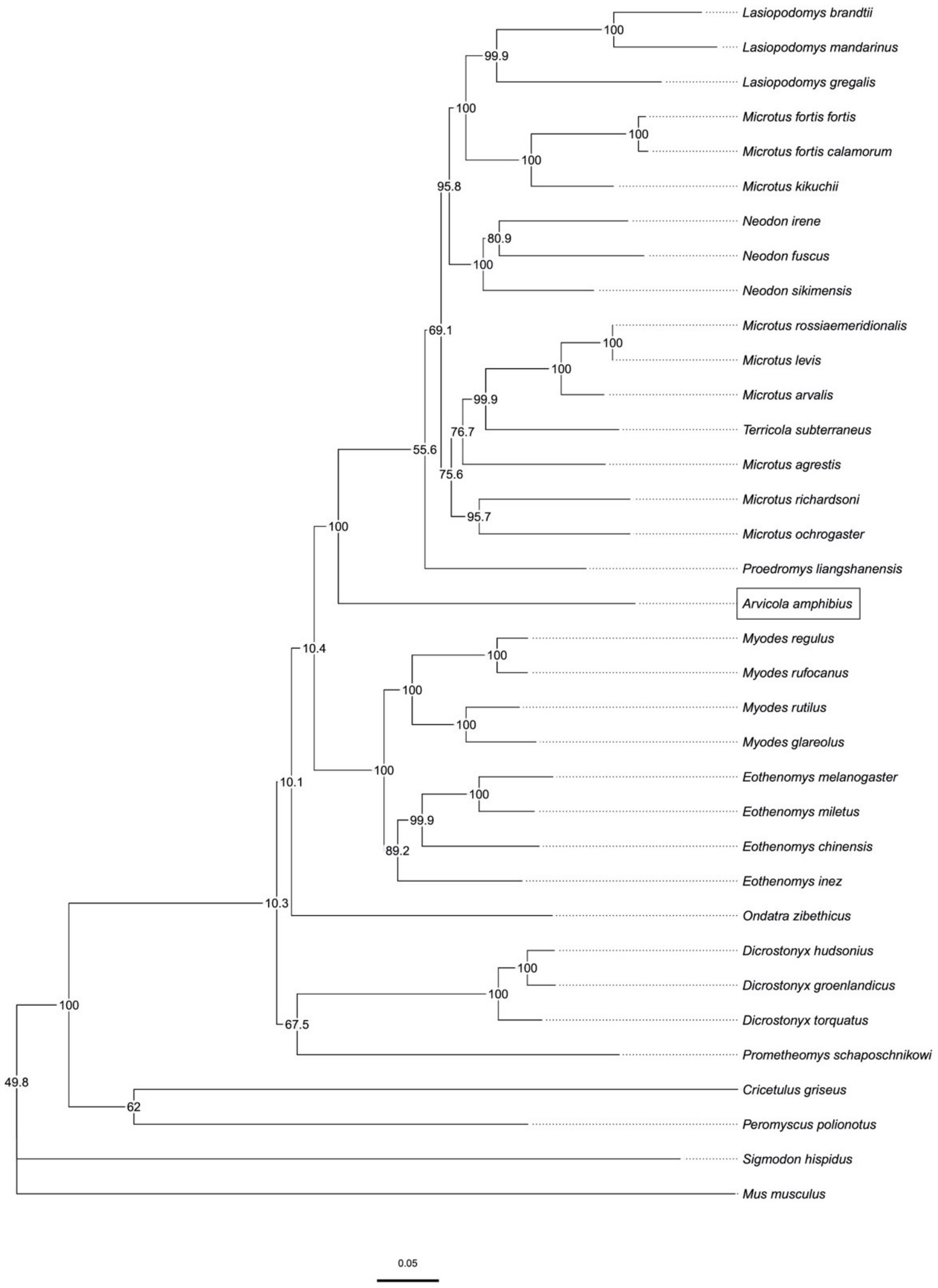

Supplementary Figure 1: ML phylogenetic tree of all available mtDNA genomes for Arvicolinae, rooted on the outgroup taxon *Mus musculus*. A single partition was considered.

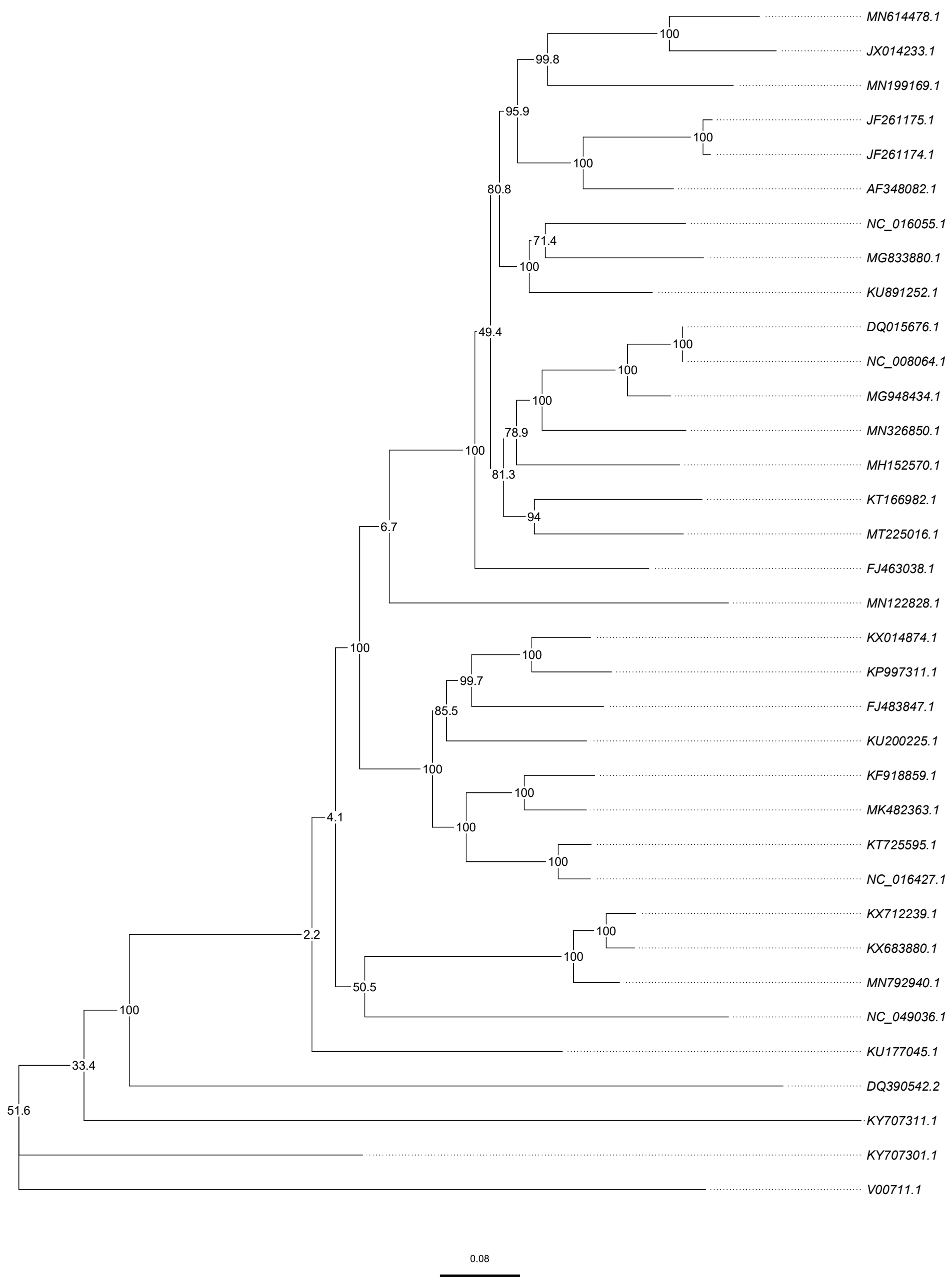

Supplementary Figure 2: ML phylogenetic tree of the 13 protein-coding genes in the mitochondrial genome for Arvicolinae, rooted on the outgroup taxon *Mus musculus*. Genes were modelled independently using PartitionFinder.
